# Publisher Correction: Agreement between commercially available ELISA and in-house Luminex SARS-CoV-2 antibody immunoassays

**DOI:** 10.1038/s41598-021-99604-2

**Published:** 2021-10-05

**Authors:** Rebeca Santano, Diana Barrios, Fàtima Crispi, Francesca Crovetto, Marta Vidal, Jordi Chi, Luis Izquierdo, Eduard Gratacós, Gemma Moncunill, Carlota Dobaño

**Affiliations:** 1 ISGlobal, Hospital Clínic, Universitat de Barcelona, Barcelona, Catalonia Spain; 2 BCNatal, Barcelona Center for Maternal-Fetal and Neonatal Medicine, Hospital Sant Joan de Déu and Hospital Clínic, IDIBAPS, Universitat de Barcelona, CIBER-ER, Barcelona, Spain

Correction to: *Scientific Reports* 10.1038/s41598-021-98296-y, published online 23 September 2021

The Funding section in the original version of this Article was omitted. The Funding section now reads:

“This study was partially funded by the KidsCorona Child and Mother COVID-19 OpenData and Biobank Initiative from Hospital Sant Joan de Déu (Stavros Niarchos Foundation, Santander Foundation and others), LaCaixa Foundation, Sant Pau Research Institute, ISGlobal and Fundació Privada Daniel Bravo Andreu, Barcelona, Spain. Development of SARS-CoV-2 reagents was partially supported by the NIAID Centers of Excellence for Influenza Research and Surveillance (CEIRS) contract HHSN272201400008C. L. I. work was supported by PID2019-110810RB-I00 grant from the Spanish Ministry of Science & Innovation.”

The original Article has been corrected.

